# Association of the Ratio of Triglycerides to High-Density Lipoprotein Cholesterol Levels with the Risk of Type 2 Diabetes: A Retrospective Cohort Study in Beijing

**DOI:** 10.1155/2021/5524728

**Published:** 2021-04-20

**Authors:** Hongzhou Liu, Shuangtong Yan, Gang Chen, Bing Li, Ling Zhao, Yajing Wang, Xiaodong Hu, Xiaomeng Jia, Jingtao Dou, Yiming Mu, Junping Wen, Zhaohui Lyu

**Affiliations:** ^1^The Department and Key Laboratory of Endocrinology and Metabolism, The First Medical Center of PLA General Hospital, Beijing 100853, China; ^2^Department of Endocrinology, The Second Medical Center & National Clinical Research Center for Geriatric Diseases, Chinese PLA General Hospital, Beijing 100853, China; ^3^Department of Endocrinology, Fujian Provincial Hospital, Key Laboratory of Endocrinology, Fujian Medical University, Fuzhou 350001, China

## Abstract

**Background:**

Previous studies have shown that the ratio of triglyceride to high-density lipoprotein cholesterol level (TG/HDL-C) is a risk factor for type 2 diabetes mellitus (T2DM). The aim of this study was to investigate the nonlinear relationship between TG/HDL-C and the incidence of T2DM in a Chinese population.

**Methods:**

We used logistic regression models to estimate odds ratios (ORs) and 95% confidence intervals (CIs) for the incidence of T2DM among 7,791 participants from the Risk Evaluation of cAncers in Chinese diabeTic Individuals: a lONgitudinal (REACTION) cohort study at baseline.

**Results:**

After adjusting for age, sex, body mass index, smoking status, alcohol intake, low-density lipoprotein cholesterol level, strenuous activity, education level, family histories of T2DM and tumors, and the presence of hypertension, tumor, stroke, and coronary heart disease, we showed that TG/HDL-C was positively associated with the incidence of T2DM at the 4-year follow-up (OR = 1.49, 95%CI = 1.26–1.78). TG/HDL-C and incidence of T2DM showed a nonlinear relationship; the inflection point of TG/HDL-C was 1.50. The ORs (95% CI) on the left and right sides of the inflection point were 2.50 (1.70–3.67) and 0.96 (0.67–1.37), respectively. After adjusting for age, sex, and body mass index (BMI) in the linear relationship, the OR of the incidence of T2DM was 1.60 (95%CI = 1.37–1.87). When the TG/HDL-C was less than 1.50 or greater than 1.76, the ORs (95% CI) were 2.41 (1.82–3.18) or 0.81 (0.53–1.25), respectively. Subgroup analysis showed no relationships of T2DM incidence with sex, BMI, family history of T2DM, or TG/HDL-C.

**Conclusion:**

TG/HDL-C is positively associated with diabetes risk. In our study, with each increasing quintile, the risk of T2DM after 4 years was 1.60 or 1.49 depending on the variables adjusted. In addition, our cohort study showed a nonlinear relationship between TG/HDL-C and T2DM incidence, with an inflection point of 1.76 or 1.50, depending on the variables adjusted. When the TG/HDL was less than 1.50, the ORs (95% CI) were 2.41 (1.82–3.18) and 2.50 (1.70–3.67). When the TG/HDL-C was greater than 1.76 or 1.50, there was no significant difference in the change in OR.

## 1. Introduction

Approximately 463 million adults (20–79 years) were living with diabetes in 2019, and this figure will increase to 700 million by 2045 [1]. Of these cases, 90% are type 2 diabetes mellitus (T2DM) [2], which can lead to complications if untreated. Acute complications include diabetic ketoacidosis, hyperosmolar hyperglycemic state, and even death [3]. Serious long-term complications include cardiovascular disease, stroke, chronic kidney disease, foot ulcers, damage to the nerves, damage to the eyes, and cognitive impairment [4]. Given the global burden of diabetes, it is important to understand the impact of modifiable risk factors on prevention.

The main cause of T2DM is insulin resistance (IR), which drives a series of metabolic processes leading to a proatherogenic lipid profile and T2DM [5, 6]. This is characterized by the decreased ability of insulin to stimulate muscle and adipose tissues to use glucose and inhibit liver glucose production and output [7]. The glucose clamp technique, which was first reported by DeFronzo et al. [8], is a classic method for assessing IR. However, it is a complex, time-consuming, and invasive method that is not feasible for routine clinical applications. Therefore, numerous indicators of IR have been evaluated, among which the ratio of triglyceride to high-density lipoprotein cholesterol levels (TG/HDL-C) was shown to be associated with IR [9–16]. However, those studies were mainly cross-sectional and did not reveal a nonlinear relationship between TG/HDL-C and T2DM incidence.

Therefore, the purpose of this retrospective cohort study was to evaluate the associations of clinical parameters related to lipid profiles with the incidence of T2DM in Chinese participants from the Risk Evaluation of cAncers in Chinese diabeTic Individuals: a lONgitudinal (REACTION) cohort study. To the best of our knowledge, few studies have assessed this nonlinear relationship among Chinese individuals with different glycemic statuses.

## 2. Methods

### 2.1. Study Participants

We used data from the REACTION cohort study, which was designed to investigate the associations of T2DM and prediabetes with the risk of cancer in a Chinese population [17]. All permanent residents aged 40 years or older of the Jingding, Laoshan, and Gucheng communities of Beijing (China) were invited to complete baseline questionnaires and medical examinations between March 2011 and December 2011.

**Figure 1:**
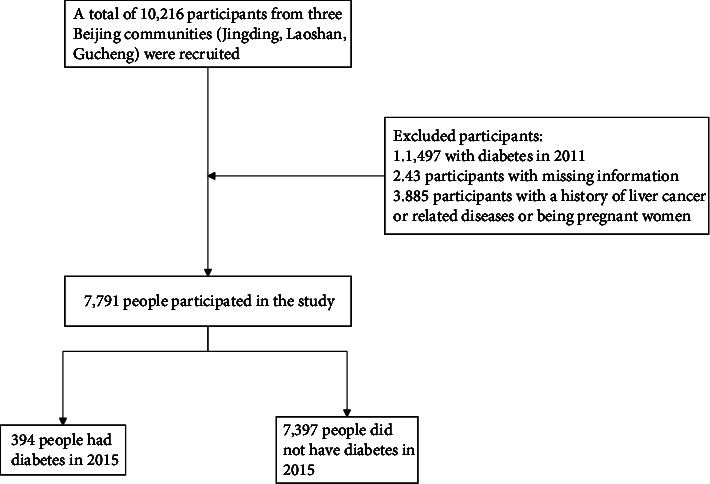
Flow chart of the study participant selection process.

A total of 10,216 individuals participated in the study. The diagnosis of T2DM was considered according to the American Diabetes Association criteria of 2003 [18]. People who have been diagnosed with T2DM or treated with hypoglycemic drugs were considered to be diabetic patients. The exclusion criteria were as follows: participants with missing information, participants with a history of liver cancer or related diseases, and pregnant women. The remaining 7,791 people participated in the study, of whom 394 people were diagnosed with T2DM in 2015 as shown in Figure 1.

### 2.2. Clinical Evaluation and Laboratory Measurements

TG/HDL-C was divided into five quintiles: <20%, 20–39%, 40–59%, 60–79%, and ≥80%. The participants underwent standardized questionnaires, body measurements, and blood collection. Trained clinicians conducted the standardized questionnaires assessing histories of tumors, stroke, coronary heart disease, hypertension, and dyslipidemia; marital status; strenuous activity; walking; education level; levels of alanine aminotransferase, aspartate aminotransferase, creatinine, fasting plasma glucose, and hemoglobin A1c; and family histories of T2DM and tumors. All data were collected according to standardized methods by the same highly trained clinicians. Physical examination included measurements of height, weight, waist circumference, hip circumference, blood pressure, and heart rate. Height was measured with bare feet to the nearest 0.01 m. Weight was measured in light clothes to the nearest 0.1 kg. Waist and hip circumferences were measured to the nearest 0.01 m by the same staff. Body mass index (BMI) was calculated as weight (kg)/height (m^2^). After resting for at least 5 min, blood pressure was measured in the seated position three times at 1 min intervals using an OMRON electronic blood pressure monitor; the average value was used for the analysis. Smoking frequency was divided into three categories: never or previous smoker, occasionally (smoking less than once a week or less than 7 cigarettes weekly), and frequently (smoking one or more cigarettes daily for at least a half year). Similarly, alcohol intake frequency was divided into three categories: never or previous drinker, occasionally (less than once a week), and frequently (more than once a week for at least a half year). Stroke, including all subtypes, was determined based on self-report, including a history of language or physical dysfunction and a history of ischemic or hemorrhagic stroke by imagological diagnosis over 24 hours. Coronary heart disease events were defined as any self-reported history of myocardial infarction, angina pectoris, or coronary revascularization.

### 2.3. Statistical Analysis

Normal distribution data were expressed as the mean ± standard deviation. Skewed distribution data were expressed as median (*P*25, *P*75). Categorical variables data were expressed as frequency or percentage. The Kolmogorov-Smirnov test was utilized for normal distribution and homogeneity test for a variance. The Kruskal-Wallis test was used in skewed distribution data to compare the differences among multiple groups of measurement data. The chi-square test was used for categorical variables. Proportional hazards models were used to calculate odds ratios (ORs) and 95% confidence intervals (95% CIs) for T2DM according to serum TG/HDL-C. Both nonadjusted and multivariate-adjusted models were used. Binary logistic models were adjusted for age, sex, BMI, smoking status, alcohol intake, low-density lipoprotein cholesterol (LDL-C), strenuous activity, education level, family histories of T2DM and tumors, and the presence of hypertension, tumors, stroke, and coronary heart disease. Trend tests were conducted using linear regression by entering the medians for each TG/HDL-C quintile in the models as continuous variables. A generalized additive model was used to evaluate the nonlinear relationship between TG/HDL-C and the incidence of T2DM. Based on the smooth curve, we further developed a two-piecewise linear regression model to determine the threshold effect, adjusting for potential confounding factors. The threshold level of TG/HDL-C was determined using a recurrence method, including selecting the turning points along predetermined intervals and selecting the turning point that produces the maximum likelihood model. The log-likelihood ratio test was used to compare the two-piecewise linear regression model with the one-line linear model. A stratified logistic regression model was used to perform subgroup analyses based on sex, BMI, and family history of T2DM. The likelihood ratio test was used to test the interactions among subgroups. For all statistical analyses, we used R version 3.4.3 (The R Foundation, Vienna, Austria). A two-way *P* value < 0.05 was considered significant.

## 3. Results

### 3.1. Baseline Characteristics of the Study Participants according to Serum TG/HDL-C

Of the 7,791 participants, 394 were diagnosed with T2DM at 4 years of follow-up. The changing tendency of blood lipids and TG/HDL-C through 4 years between T2DM patients and non-T2DM controls is shown in Supplementary Table 1.  Table 1 lists the baseline characteristics of all participants. The mean age was 56.03 ± 7.82 years, and one-third (2,613, 33.54%) of the participants were male. The mean TG/HDL-C was 1.10 ± 0.62. Participants with higher TG/HDL-C values were more likely to be male and smokers and to have hypertension, stroke, hyperlipidemia, lower walking frequency, and a family history of T2DM. In addition, serum TG/HDL-C was directly proportional to systolic and diastolic blood pressure, BMI, waist circumference, hip circumference, presence of fatty liver, and the levels of alanine aminotransferase, creatinine, total cholesterol, TGs, LDL-C, fasting plasma glucose, and hemoglobin A1c, but inversely proportional to the HDL-C level.

### 3.2. Association between Serum TG/HDL-C and T2DM Incidence

Table 2 shows the ORs and 95% CIs for developing T2DM according to TG/HDL-C quintile. In the nonadjusted model, the risk of T2DM increased as the TG/HDL-C increased by 20% (*P* for trend <0.01). Participants whose TG/HDL-C was between the highest and lowest quintile had a nearly fourfold increased risk of developing T2DM (OR = 3.71, 95%CI = 2.51–5.51). After adjusting for age, sex, BMI, histories of hypertension, tumors, stroke, and coronary heart disease, smoking status, alcohol intake, LDL-C, strenuous activity, education level, and family histories of T2DM and tumors, the ORs (95% CI) were 1.34 (0.82–2.20) (*P* = 0.24), 1.55 (0.96–2.51) (*P* = 0.07), 2.23 (1.41–3.53) (*P* < 0.01), and 2.42 (1.53–3.84) (*P* < 0.01) for TG/HDL-C quintiles 2–5, respectively (*P* for trend <0.01).

### 3.3. Threshold Effect Analysis of TG/HDL-C on the Incidence of T2DM

**Figure 2:**
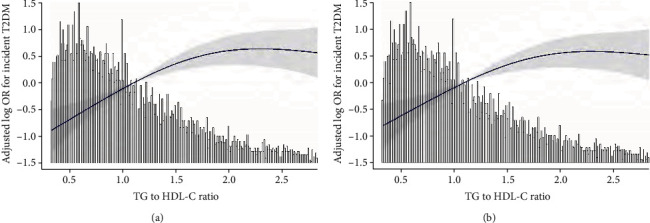
(a) Threshold effect analysis of TG/HDL-C on the incidence of T2DM in the REACTION study. Notes: adjusted for age, sex, and body mass index. (b) Threshold effect analysis of TG/HDL-C on the incidence of T2DM in the REACTION study. Notes: adjusted for age, sex, body mass index, histories of hypertension, tumor, stroke, and coronary heart disease, smoking status, alcohol intake, low-density lipoprotein cholesterol level, strenuous activity, education level, and family histories of T2DM and tumors.

To evaluate whether a dose-response relationship exists between TG/HDL-C and the incidence of T2DM, we used a smooth function analysis. After adjusting for age, sex, and BMI, a nonlinear relationship between TG/HDL-C and T2DM was observed (Figure 2(a)). The risk of developing T2DM was positively correlated with TG/HDL-C until the ratio reached 1.76 (OR = 2.41, 95%CI = 1.82–3.18, *P* < 0.01). However, at TG/HDL − C > 1.76, the OR for T2DM was 0.81 (95%CI = 0.53–1.25), indicating that the risk of T2DM did not increase significantly with increasing TG/HDL-C (*P* = 0.35) (Table 3).

After adjusting for age, sex, body mass index, histories of hypertension, tumors, stroke, and coronary heart disease, smoking status, alcohol intake, LDL-C level, strenuous activity, education level, and family histories of T2DM and tumors, a nonlinear relationship between TG/HDL-C and T2DM incidence was observed (Figure 2(b)). The risk of T2DM was positively correlated with serum TG/HDL-C until the ratio reached 1.50 (OR = 2.50, 95%CI = 1.70–3.67, *P* < 0.01). However, when TG/HDL-C exceeded 1.50, the OR for developing T2DM was 0.96 (95%CI = 0.67–1.37), indicating that the risk of T2DM did not increase significantly as TG/HDL-C increased (*P* = 0.82) (Table 4).

### 3.4. Subgroup Analyses

To explore whether the correlation between TG/HDL-C and T2DM incidence exists among different subgroups, we conducted stratified analyses and interactive analyses (Table 5). The data showed that age played an important role in the association between TG/HDL-C and incidence of T2DM (*P* for interaction <0.01). The associations in the top four quintiles of TG/HDL-C were stronger for participants aged <60 years (quintile 2, 1.67 (0.91–0.67); quintile 3, 2.59 (1.47–4.56); quintile 4, 3.28 (1.88–5.70); and quintile 5, 4.50 (2.61–7.75) vs. quartile 1, 1.00; *P* for trend <0.01). No significant associations were observed among the other subgroups.

## 4. Discussion

In this cohort study, TG/HDL-C was shown to be associated with an elevated risk of T2DM, independent of age, sex, body mass index, histories of hypertension, tumor, stroke, and coronary heart disease, smoking status, alcohol intake, LDL-C, strenuous activity, education level, and family histories of T2DM and tumors. We showed a nonlinear relationship between serum TG/HDL-C and risk of T2DM; in that, the risk of T2DM after 4 years increased significantly with an increase in TG/HDL-C when the ratio was less than 1.76 or 1.50, depending on the variables adjusted.

The diagnosis of IR is based on simultaneous measurements of glucose and insulin. The classic method for IR measurement is the metabolic euglycemic clamp, but this method is laborious and expensive and thus is used mainly for research purposes. IR affects the metabolism of TGs, HDL-C, and LDL-C [6]. High TG and low HDL-C levels are associated with IR and T2DM [19], but TG and HDL-C levels alone are weaker risk factors compared with the TG/HDL-C [9, 20]. McLaughlin et al. [21] were the first to demonstrate the clinical utility of TG/HDL-C for identifying healthy Caucasians with IR, including 258 healthy nondiabetic individuals. The results showed that TG/HDL-C was closely related to specific indicators of insulin-mediated glucose disposal and the fasting plasma insulin concentration. This ratio has been widely used to assess the associations between IR and various clinical syndromes. Most previous studies on the relationship between TG/HDL-C and the incidence of T2DM reported a positive association [10, 16, 22], and similar results can be found for type 1 diabetes mellitus [23]. Our findings on TG/HDL-C are consistent with those studies. As shown in Table 2, the incidence of T2DM increased with increasing TG/HDL-C. In the lowest TG/HDL-C quintile, the OR (95% CI) for developing T2DM after 4 years of follow-up was 2.88 (1.92–4.31) or 2.42 (1.53–3.84) depending on the variables adjusted. Previous studies have also explored the relationship between TG/HDL-C and the incidence of T2DM. Cheng et al. [24] also showed a nonlinear relationship between TG/HDL-C and the overall risk of T2DM (*P* < 0.01). The risk of T2DM continued to increase with increasing TG/HDL-C, with a gradual increase as TG/HDL-C exceeded 2.5 in males. Our study showed that when TG/HDL was <1.76 or <1.50, depending on the variables adjusted, the OR (95% CI) was 2.41 (1.82–3.18) or 2.50 (1.70–3.67), respectively. When TG/HDL was >1.76 or >1.50, there was no statistical difference in the change in OR.

Lipid and glucose metabolism is affected by many factors. We analyzed whether there were differences in the relationship according to different subgroups. The associations between TG/HDL-C and risk of T2DM were significant only with age < 60 years, which was similar with the result of Zhang et al. [11]. As compared with young people, the anabolism of older people is significantly lower [25]. Pramfalk et al. [26] reported that despite no significant sex difference in the plasma total cholesterol (TC) level, men had significantly higher fasting plasma TG levels, while women had lower plasma LDL-C and higher HDL-C. However, the *P* value for interaction was 0.53 in our subgroup analysis, and there was no effect of sex on TG/HDL-C or T2DM incidence, suggesting that the association between TG/HDL-C and T2DM incidence after 4 years is similar between the sexes. The effect of sex on blood lipid metabolism is still controversial, and we will further explore the influence of gender on the relationship between TG/HDL-C and the incidence of diabetes in the next follow-up study. Iwani et al. [27] showed that TG/HDL-C is significantly associated with IR. TG/HDL-C is an inexpensive predictor of IR and may be a useful tool to identify high-risk individuals for early intervention, thereby preventing or delaying the development of IR-associated diseases such as T2DM. The interaction of this study showed that in people with BMI less than 25 kg/m^2^ and BMI greater than 25 kg/m^2^, the incidence of T2DM increased as the TG/HDL-Co increased. This indicated that at different BMIs, the ability of TG/HDL-C to predict the incidence of diabetes is the same. As they share genetic and environmental factors with T2DM patients, the first-degree relatives of T2DM patients show early signs of metabolic abnormalities [28]. In our model, there was no interaction between family history of T2DM and TG/HDL-C; that is, the relationship between T2DM incidence and TG/HDL-C was independent of a family history of T2DM. We have only been followed up for 4 years, and the incidence of T2DM is only 5%. So it may lead to the influence of the family history of diabetes on the incidence of T2DM. We will continue to analyze the impact of family history of T2DM on the incidence of diabetes after the next follow-up.

## 5. Limitations

There are potential limitations of this study to note. First, T2DM is associated with region and ethnicity. As this cohort study was conducted in Beijing, our findings may not be applicable to other regions, ethnicities, or special groups such as children and pregnant women. Second, the presence of T2DM was reported only at the 4-year follow-up, while the specific date of diagnosis was not recorded; thus, we could only perform logistic regression, which is weaker than Cox regression. The date of T2DM diagnosis during the next follow-up needs to be determined to obtain more information for analysis.

## 6. Conclusions

TG/HDL-C was positively associated with diabetes risk. In our study, for every increase in TG/HDL-C quintile, the risk of T2DM after 4 years was 1.60 or 1.49 depending on the variables adjusted. In addition, a nonlinear relationship between TG/HDL-C and T2DM incidence was found in our cohort study. The inflection point of TG/HDL-C was 1.76 or 1.50, depending on the variables adjusted. When the TG/HDL-C was less than 1.76 or 1.50, the ORs (95% CI) were 2.41 (1.82–3.18) and 2.50 (1.70–3.67), respectively. When the TG/HDL-C was greater than 1.76 or 1.50, there was no statistical difference in the change in OR.

## Figures and Tables

**Table 1:** Baseline characteristics of the participants in the REACTION study according to serum TG/HDL-C.

Variable	All participants	TG/HDL-C					*P* value
		Q1 (1558)	Q2 (1557)	Q3 (1560)	Q4 (1557)	Q5 (1559)	
Age (years)	56.03 ± 7.82	55.15 ± 7.88	56.22 ± 8.10	56.36 ± 7.64	56.21 ± 7.80	56.24 ± 7.60	<0.01
Male	2613 (33.54%)	419 (26.89%)	493 (31.66%)	504 (32.31%)	591 (37.96%)	606 (38.87%)	<0.01
TG/HDL-C	1.10 ± 0.62	0.46 ± 0.07	0.68 ± 0.07	0.94 ± 0.08	1.31 ± 0.14	2.12 ± 0.44	<0.01
SBP (mmHg)	131.22 ± 16.24	127.98 ± 16.67	129.42 ± 15.86	131.95 ± 16.20	132.76 ± 16.02	134.02 ± 15.68	<0.01
DBP (mmHg)	75.84 ± 9.52	73.47 ± 9.13	74.74 ± 9.5	76.21 ± 9.43	76.99 ± 9.56	77.78 ± 9.35	<0.01
Heart rate	78.65 ± 11.29	77.93 ± 11.25	78.06 ± 11.26	78.72 ± 11.41	79.22 ± 11.38	79.32 ± 11.07	<0.01
Height (kg)	161.12 ± 7.76	160.52 ± 7.36	160.61 ± 7.67	160.81 ± 7.64	161.8 ± 7.98	161.87 ± 8.04	<0.01
Weight (cm)	66.96 ± 10.84	62.15 ± 9.64	65.23 ± 10.17	67.26 ± 10.29	69.60 ± 10.95	70.55 ± 10.96	0.31
BMI (kg/m^2^)	25.74 ± 3.41	24.09 ± 3.16	25.26 ± 3.32	25.97 ± 3.30	26.53 ± 3.30	26.86 ± 3.25	<0.01
WC (cm)	83.77 ± 17.14	78.81 ± 8.15	82.12 ± 8.47	84.44 ± 24.72	86.01 ± 8.30	87.46 ± 24.59	<0.01
HC (cm)	94.71 ± 16.07	92.06 ± 6.44	93.68 ± 6.54	95.46 ± 23.93	95.69 ± 7.04	96.66 ± 23.91	<0.01
ALT (U/L)	18.30 (14.20-24.70)	15.90 (12.80, 20.70)	17.10 (13.30, 22.60)	18.50 (14.60, 24.40)	19.50 (15.20, 27.10)	21.00 (16.30, 28.70)	<0.01
AST (U/L)	19.60 (16.70-23.20)	19.50 (16.90-22.40)	19.30 (16.50-22.80)	19.80 (16.90-23.40)	19.70 (16.80-23.70)	19.80 (16.80-23.60)	0.051
Cr (mmol/L)	67.18 ± 13.97	64.77 ± 12.82	66.00 ± 13.55	67.27 ± 14.15	68.74 ± 14.58	69.13 ± 14.24	<0.01
TC (mmol/L)	5.28 ± 1.53	5.10 ± 1.44	5.23 ± 1.84	5.29 ± 0.95	5.32 ± 1.54	5.43 ± 1.71	<0.01
TG (mmol/L)	1.45 ± 0.63	0.80 ± 0.15	1.06 ± 0.19	1.32 ± 0.22	1.66 ± 0.28	2.39 ± 0.54	<0.01
HDL-C (mmol/L)	1.43 ± 0.32	1.76 ± 0.29	1.56 ± 0.26	1.41 ± 0.22	1.28 ± 0.20	1.13 ± 0.18	<0.01
LDL-C (mmol/L)	3.26 ± 0.80	2.96 ± 0.71	3.19 ± 0.78	3.34 ± 0.77	3.39 ± 0.77	3.44 ± 0.86	<0.01
FPG	5.43 ± 0.53	5.31 ± 0.49	5.41 ± 0.53	5.42 ± 0.53	5.46 ± 0.54	5.53 ± 0.56	<0.01
HbA1C (%)	5.87 ± 0.62	5.73 ± 0.52	5.83 ± 0.55	5.86 ± 0.57	5.90 ± 0.63	6.02 ± 0.77	<0.01
Hypertension	2167 (27.82%)	307 (19.72%)	401 (25.75%)	432 (27.69%)	494 (31.73%)	533 (34.19%)	<0.01
Tumor	156 (2.00%)	22 (1.41%)	34 (2.18%)	34 (2.18%)	26 (1.67%)	40 (2.57%)	0.15
Stroke	189 (2.43%)	23 (1.48%)	34 (2.18%)	43 (2.76%)	39 (2.50%)	50 (3.21%)	0.03
CHD	474 (6.08%)	82 (5.27%)	91 (5.84%)	94 (6.03%)	97 (6.23%)	110 (7.06%)	0.33
Hyperlipidemia	1067 (13.70%)	107 (6.87%)	152 (9.76%)	204 (13.08%)	234 (15.03%)	370 (23.73%)	<0.01
Marital status							0.67
Married	7316 (93.92%)	1445 (92.81%)	1465 (94.09%)	1469 (94.17%)	1455 (93.45%)	1482 (95.06%)	
Unmarried	29 (0.37%)	5 (0.32%)	6 (0.39%)	6 (0.38%)	6 (0.39%)	6 (0.38%)	
Widowed	262 (3.36%)	65 (4.17%)	48 (3.08%)	53 (3.40%)	54 (3.47%)	42 (2.69%)	
Divorced	179 (2.30%)	40 (2.57%)	38 (2.44%)	31 (1.99%)	41 (2.63%)	29 (1.86%)	
Else	4 (0.05%)	2 (0.13%)	0 (0.00%)	1 (0.06%)	1 (0.06%)	0 (0.00%)	
Education level							0.048
Illiteracy	77 (0.99%)	17 (1.09%)	17 (1.09%)	12 (0.77%)	16 (1.03%)	15 (0.96%)	
Primary school	370 (4.75%)	68 (4.37%)	94 (6.05%)	79 (5.07%)	72 (4.63%)	57 (3.66%)	
Junior high school	2547 (32.73%)	456 (29.31%)	496 (31.90%)	535 (34.32%)	525 (33.74%)	535 (34.36%)	
High school or technical secondary school	3460 (44.46%)	729 (46.85%)	687 (44.18%)	682 (43.75%)	689 (44.28%)	673 (43.22%)	
College or above	1329 (17.08%)	286 (18.38%)	261 (16.78%)	251 (16.10%)	254 (16.32%)	277 (17.79%)	
Smoking (%)							<0.01
Never	6370 (81.88%)	1358 (87.22%)	1291 (83.18%)	1293 (83.04%)	1226 (78.79%)	1202 (77.15%)	
Occasionally	169 (2.17%)	27 (1.73%)	32 (2.06%)	33 (2.12%)	40 (2.57%)	37 (2.37%)	
Frequently	1241 (15.95%)	172 (11.05%)	229 (14.76%)	231 (14.84%)	290 (18.64%)	319 (20.47%)	
Alcohol intake							0.78
Never	5515 (70.85%)	1120 (71.89%)	1095 (70.46%)	1129 (72.37%)	1083 (69.65%)	1088 (69.88%)	
Occasionally	1470 (18.88%)	285 (18.29%)	302 (19.43%)	277 (17.76%)	304 (19.55%)	302 (19.40%)	
Frequently	799 (10.26%)	153 (9.82%)	157 (10.10%)	154 (9.87%)	168 (10.80%)	167 (10.73%)	
Strenuous activity							0.24
Yes	222 (2.86%)	51 (3.28%)	32 (2.07%)	47 (3.02%)	50 (3.22%)	42 (2.70%)	
No	7543 (97.14%)	1502 (96.72%)	1517 (97.93%)	1510 (96.98%)	1502 (96.78%)	1512 (97.30%)	
Walking							<0.01
Yes	6742 (86.80%)	1369 (88.15%)	1376 (88.83%)	1336 (85.81%)	1349 (86.92%)	1312 (84.32%)	
No	1025 (13.20%)	184 (11.85%)	173 (11.17%)	221 (14.19%)	203 (13.08%)	244 (15.68%)	
Family history of T2DM	1916 (24.61%)	363 (23.30%)	381 (24.49%)	373 (23.96%)	374 (24.04%)	425 (27.28%)	0.09
Family history of tumors	1655 (21.26%)	340 (21.82%)	319 (20.50%)	325 (20.87%)	340 (21.85%)	331 (21.25%)	0.86

TG: triglyceride; HDL-C: high-density lipoprotein cholesterol; SBP: systolic blood pressure; DBP: diastolic blood pressure; BMI: body mass index; WC: waist circumference; HC: hip circumference; ALT: alanine aminotransferase; AST: aspartate aminotransferase; Cr: creatinine; TC: total cholesterol; TG: triglyceride; HDL-C: high-density lipoprotein cholesterol; LDL-C: low-density lipoprotein cholesterol; FPG: fasting plasma glucose; HbA1c: hemoglobin A1c; CHD: coronary heart disease.

**Table 2:** Association between serum TG/HDL-C and the incidence of T2DM in the REACTION study.

	Crude	Model I	Model II
TG/HDL-C	1.78 (1.53–2.06) <0.01	1.60 (1.37–1.87) <0.01	1.49 (1.26–1.78) <0.01
TG/HDL-C quintile			
Q1	1	1	1
Q2	1.53 (0.98–2.39) 0.06	1.36 (0.87–2.13) 0.17	1.34 (0.82–2.20) 0.24
Q3	1.98 (1.29–3.03) <0.01	1.66 (1.08–2.55) 0.02	1.55 (0.96–2.51) 0.07
Q4	2.94 (1.96–4.39) <0.01	2.32 (1.53–3.50) <0.01	2.23 (1.41–3.53) <0.01
Q5	3.71 (2.51–5.51) <0.01	2.88 (1.92–4.31) <0.01	2.42 (1.53–3.84) <0.01
*P* for trend	<0.01	<0.01	<0.01

Data are ORs (95% CI). Model I was adjusted for age, sex, and body mass index. Model II was adjusted for the variables in model I plus histories of hypertension, tumors, stroke, and coronary heart disease, smoking status, alcohol intake, low-density lipoprotein cholesterol level, strenuous activity, education level, and family histories of T2DM and tumors.

**Table 3:** Threshold effect analysis of TG/HDL-C on the incidence of type 2 diabetes mellitus in the REACTION study.

Outcomes	OR (95% CI)	*P* value
One-line linear regression model	1.60 (1.37–1.87)	<0.01
Two-piecewise linear regression model		
<1.76	2.41 (1.82–3.18)	<0.01
>1.76	0.81 (0.53–1.25)	0.35
Log-likelihood ratio test		<0.01

Adjusted for age, sex, and body mass index.

**Table 4:** Threshold effect analysis of TG/HDL-C on the incidence of type 2 diabetes mellitus in the REACTION study.

Outcome	OR (95% CI)	*P* value
One-line linear regression model	1.49 (1.26–1.78)	<0.01
Two-piecewise linear regression model		
<1.50	2.50 (1.70–3.67)	<0.01
>1.50	0.96 (0.67–1.37)	0.82
Log-likelihood ratio test		<0.01

Adjusted for age, sex, body mass index, histories of hypertension, tumor, stroke, and coronary heart disease, smoking status, alcohol intake, low-density lipoprotein cholesterol level, strenuous activity, education level, and family histories of T2DM and tumors.

**Table 5:** Subgroup analyses of the association between TG/HDL-C and incidence of type 2 diabetes mellitus in the REACTION study.

Confounding factor	Serum TG/HDL-C quintile					*P* for trend	*P* for interaction
	Q1	Q2	Q3	Q4	Q5		
Age							0.01
<60 years	1	1.67 (0.91, 3.07) 0.10	2.59 (1.47, 4.56) <0.01	3.28 (1.88, 5.70) <0.01	4.50 (2.61, 7.75) <0.01	<0.01	
>60 years	1	0.96 (0.48, 1.92) 0.90	0.74 (0.36, 1.54) 0.43	1.30 (0.67, 2.54) 0.44	1.08 (0.55, 2.12) 0.82	0.50	
Sex							0.53
Male	1	1.51 (0.69–3.31) 0.30	1.48 (0.68–3.22) 0.33	1.65 (0.78–3.50) 0.19	2.19 (1.05–4.57) 0.04	0.03	
Female	1	1.21 (0.69–2.11) 0.50	1.75 (1.04–2.96) 0.03	2.61 (1.58–4.31) <0.01	2.97 (1.81–4.88) <0.01	<0.01	
BMI							0.83
<25	1	1.43 (0.73–2.79) 0.30	1.85 (0.95–3.60) 0.07	2.39 (1.22–4.67) 0.01	3.71 (1.97–6.99) <0.01	<0.01	
≥25	1	1.26 (0.68–2.35) 0.46	1.55 (0.86–2.79) 0.14	2.19 (1.25–3.83) 0.01	2.40 (1.38–4.18) <0.01	<0.01	
Family history of T2DM							0.20
Yes	1	1.09 (0.50–2.34) 0.8309	1.76 (0.87–3.6) 0.1155	2.79 (1.42–5.47) 0.0029	2.18 (1.09–4.34) 0.0267	0.01	
No	1	1.55 (0.88–2.73) 0.1281	1.69 (0.97–2.93) 0.0639	2.18 (1.28–3.72) 0.0041	3.31 (1.98–5.55) <0.0001	<0.01	

Adjusted for age, sex, body mass index, histories of hypertension, tumors, stroke, and coronary heart disease, smoking status, alcohol intake, low-density lipoprotein cholesterol level, strenuous activity, education level, and family histories of T2DM and tumors.

## Data Availability

The data and analytical methods of this study are available from the corresponding author upon reasonable request.

## Supplementary Materials

Supplementary Materials During the 4 years, the level of blood lipid and TG/HDL-C changed significantly (*P* < 0.05). (Supplementary Table 1) We are all grouped according to the data of the first time.
