# Supplementary material for: Association of the Ratio of Triglycerides to High-Density Lipoprotein Cholesterol Levels with the Risk of Type 2 Diabetes: A Retrospective Cohort Study in Beijing
Source: J Diabetes Res. 2021 Apr 20;2021:5524728. doi: 10.1155/2021/5524728 (PMC8081643; doi:10.1155/2021/5524728)
Supplement: Supplementary Materials — During the 4 years, the level of blood lipid and TG/HDL-C changed significantly (P < 0.05). (Supplementary Table 1) We are all grouped according to the data of the first time. [file 5524728.f1.docx]

Supplement table 1. changing tendency of blood lipids and TG/HDL-C through 4 years between non-T_2_DM patients and T_2_DM controls.

|  | non-T_2_DM | | |  | T_2_DM | | |
| --- | --- | --- | --- | --- | --- | --- | --- |
|  | 2011 | 2015 | *P* |  | 2011 | 2015 | *P* |
| TC (mmol/L) | 5.26±1.38 | 4.93±0.96 | <0.01 |  | 5.55±3.42 | 4.65±0.97 | <0.01 |
| TG (mmol/L) | 1.43±0.63 | 1.52±0.85 | <0.01 |  | 1.68±0.66 | 1.60±1.14 | <0.01 |
| HDL-C (mmol/L) | 1.43±0.32 | 1.44±0.35 | <0.01 |  | 1.33±0.27 | 1.37±0.32 | <0.01 |
| LDL-C (mmol/L) | 3.26±0.79 | 3.18±0.85 | <0.01 |  | 3.42±0.85 | 2.93±0.83 | <0.01 |
| TG/HDL-C | 0.92 (0.61, 1.40) | 0.93 (0.63, 1.43) | <0.01 |  | 1.26 (0.83, 1.76) | 0.99 (0.68, 1.61) | <0.01 |
